# Morphometric analysis of young petiole galls on the narrow-leaf cottonwood, *Populus angustifolia*, by the sugarbeet root aphid, *Pemphigus betae*

**DOI:** 10.1007/s00709-015-0937-8

**Published:** 2016-01-06

**Authors:** Ryan A. Richardson, Mélanie Body, Michele R. Warmund, Jack C. Schultz, Heidi M. Appel

**Affiliations:** 1 Division of Plant Sciences, Christopher S. Bond Life Sciences Center, University of Missouri, 1201 Rollins Street, Columbia, MO 65211 USA; 2 Division of Plant Sciences, Department of Horticulture, University of Missouri, 1-31 Agriculture Building, Rollins Street, Columbia, MO 65211 USA

**Keywords:** Gall-inducing insect, Galling aphid, Morphological alterations, Nutritive tissue, Microscopy

## Abstract

An insect-induced gall is a highly specialized structure resulting from atypical development of plant tissue induced by a reaction to the presence and activity of an insect. The insect induces a differentiation of tissues with features and functions of an ectopic organ, providing nutrition and protection to the galling insect from natural enemies and environmental stresses. In this anatomical and cytological study, we characterized how the gall-inducing aphid *Pemphigus betae* reshapes the leaf morphology of the narrow-leaf cottonwood *Populus angustifolia* to form a leaf fold gall. Young galls displayed a bend on one side of the midvein toward the center of the leaf and back to create a fold on the abaxial side of the leaf. This fold was formed abaxially by periclinal and anticlinal divisions, effectively eliminating intercellular spaces from the spongy parenchyma. Galls at this stage exhibited both cell hypertrophy and tissue hyperplasia. Cells on the adaxial surface were more numerous and smaller than cells near the abaxial surface were, creating the large fold that surrounds the insect. Mesophyll cells exhibited some features typical of nutritive cells induced by other galling insects, including conspicuous nucleolus, reduced and fragmented vacuole, smaller and degraded chloroplasts, and dense cytoplasm compared to ungalled tissue. Even though aphids feed on the contents of phloem and do not directly consume the gall tissue, they induce changes in the plant vascular system, which lead to nutrient accumulation to support the growing aphid numbers in mature galls.

## Introduction

Galls are highly specialized structures arising from atypical development of plant tissue induced by another organism. Many different kinds of organisms can induce galls on plants, including viruses, fungi, bacteria, nematodes, mites, and insects (Redfern 2011). However, insects make galls that are more structurally consistent and diverse than those made by all other gall-inducing organisms (Imms 1947; Price et al. 1987). Galling has evolved repeatedly among and within insect orders: Hymenoptera, Diptera, Hemiptera, Lepidoptera, Coleoptera, and Thysanoptera (Stone and Schönrogge 2003). An estimated 15,000 insect species manipulate the development of their host plants in a species-specific manner to generate galls within which the insect feeds. Insect galls are distinguished from other insect-generated shelters (such as rolled leaves or leaf mines) by the active differentiation and growth of plant tissues with features of a novel organ (Mani 1964; Stone and Schönrogge 2003; Shorthouse et al. 2005; Giron et al. 2015). These structures are thought to provide adaptive advantages to gall feeders of enhanced nutrition and protection of the galling insect against natural enemies and environmental stresses (Mani 1964; Price et al. 1987; Hartley and Lawton 1992; Hartley 1998; Nyman and Julkunen-Tiitto 2000; Stone et al. 2002; Nakamura et al. 2003; Stone and Schönrogge 2003; Allison and Schultz 2005; Motta et al. 2005; Ikai and Hijii 2007; Diamond et al. 2008; Formiga et al. 2009; Compson et al. 2011; Formiga and Isaias 2011; Nabity et al. 2013).

Gall formation is a complex and close interaction between the insect and the host plant resulting from molecular cross-talk between two independent genomes. The inducer manipulates the host plant signaling by injecting effectors (small molecules that alter host cell structure and function and modulate plant response) into the wound while initiating interaction with the host (during feeding and/or oviposition depending on insect species) to redirect normal plant development (Chen et al. 2010; Hogenhout and Bos 2011; Giron et al. 2015). The chemical identity and mode of action of the inducing compounds in these secretions, and the plant developmental pathways that they affect, remain unclear (Giron et al. 2015). Unlike the host genetic transformation used by *Agrobacterium tumefaciens* to cause crown gall on plants, insect galls are not thought to involve host genetic transformation because insect gall development stops if the insect is removed. Diverse chemical signals have been proposed in insect gall systems, including phytohormones (especially plant growth factors: auxins and/or cytokinins) (Cornell 1983; Shorthouse and Rohfritsch 1992; Suzuki et al. 2014; Tooker and Helms 2014), amino acids (Stone and Schönrogge 2003), proteins (Higton and Mabberly 1994), mutualistic viruses (Cornell 1983), or bacterial symbionts (Yamaguchi et al. 2012). Whatever their nature, these chemical signals generate galls with morphological phenotypes characteristic of each inducing species (Rohfritsch 1992; Williams 1994; Crespi and Worobey 1998; Stone and Schönrogge 2003). Some plant species support a comparatively rich fauna (two or more species) of insect galls, each with different morphological features (Formiga et al. 2015). For example, up to 70 distinct gall structures may be present on a single oak, each caused by a different insect species (Stone and Schönrogge 2003; Stone G, personal communication). Although more rare in nature, the same insect species can induce morphologically similar galls in different host plants, which is evidence that galling insects can play a major role in determining gall morphology (Price et al. 1987; Stone and Schönrogge 2003; Muñoz-Viveros et al. 2014). In some lineages, especially gall wasps (Hymenoptera, Cynipidae) and gall midges (Diptera: Cecidomyiidae), gall formation involves elaborate complex external structures, including extrafloral nectaries, hair, spines, and sticky resins (Stone and Schönrogge 2003). Thus, the insect gall phenotype is a product of a chemical communication between the host plant and the gall-inducer and is under the influence of both the insect and the plant genotypes. Indeed, galls are commonly considered to be the extended phenotype of the gall inducer (Dawkins 1982), with the developmental program of plant cells altered toward new shape and function.

Gall-inducing insects have different ways of harvesting the plant food. Some gallers are biting/chewing insects (caterpillar-like) that consume plant cells by macerating entire tissues and rupturing cells with their mandibles in the process, whereas other gallers have piercing/sucking mouthparts (aphid-like) and penetrate plant tissue with their stylets allowing them to reach the vascular elements to feed on plant sap (Forbes 1977; Schoonhoven et al. 2005; Chapman 2013). Depending on these feeding habits, specialized nutritive tissues may differentiate. Galls, especially those induced by Cecidomyiidae and Cynipidae, usually contain a highly differentiated nutritive layer that lines the central chamber and is consumed by the larva during its development (Rohfritsch 1977; Bronner 1992). However, other galling-insects, such as psyllids, aphids, and their relatives, induce limited changes in host tissue that is called a nutritive-like layer (Álvarez et al. 2009; Oliveira and Isaias 2010b; Isaias and Oliveira 2012; Carneiro and Isaias 2015a, b). The nutritive cells usually display a common set of cytological features, even though other aspects of gall morphology and organization can vary widely (Muñoz-Viveros et al. 2014). The chlorenchyma cells within the nutritive tissue are generally homogenous and usually includes a large nucleus, conspicuous nucleolus, high enzymatic activity, RNA richness, fragmented vacuole, numerous mitochondria, a dense/abundant cytoplasm, and the accumulation of carbohydrates (and lipids in some systems) (Bronner 1992). These cells also have thin walls and reduced intercellular spaces that are characteristic of young, fast-growing tissues (Castro et al. 2012; Vecchi et al. 2013; Carneiro and Isaias 2015a). The lack of intercellular spaces indicates the occurrence of little gas exchange and consequent reduced photosynthetic metabolism (Carneiro and Isaias 2015a). Chloroplasts and mitochondria are numerous and poorly differentiated, often leading to photosynthesis-deficient cells within the galls (Bronner 1992; Huang et al. 2014; Carneiro and Isaias 2015a). Understanding these insect-induced cytological changes may help elucidate how the insect induces gall formation.

In this study, we focus on aphid galls formed on narrowleaf cottonwood trees because they have ecological and genetic resources that will facilitate future mechanistic studies of gall formation. The narrowleaf cottonwood, *Populus angustifolia*, is a foundation tree species for about 700 insect species, soil microbial communities, lichens, fungi, beavers, and birds (Whitham et al. 2006, 2008). The presence or absence of the leaf-galling aphid *Pemphigus betae* is determined by susceptible or resistant poplar genotypes and affects other trophic levels by altering the composition of a diverse community of fungi, insects, spiders, and avian predators (Whitham et al. 2006, 2008). The *Pemphigus* aphid and other communities of arthropods on narrowleaf cottonwood alter the chemistry (such as sugar and condensed tannin contents) within the tree which in turn affects other species that depend on the tree, leading to major community and ecosystem consequences (Larson and Whitham 1991; Whitham et al. 2006).

This *Populus-Pemphigus* system has been studied by ecologists for over three decades to understand the interactions among all species in the ecosystem, but the mechanism of gall formation is unknown. As its common name implies, the sugarbeet root aphid uses other plant species as secondary hosts, including sugarbeet, an important crop for sucrose production in the northern USA (Larson and Whitham 1991). *P. betae* causes significant reductions in sugarbeet yield and reduces sucrose quality. For example, in 1989, a *Pemphigus* infestation reduced the sugar content and recoverable sugar by 64 and 73 %, respectively, resulting in a $3,000,000 loss or about $925 per infested hectare (Hutchinson and Campbell 1994). Thus, an understanding of the mechanism of gall development by *P. betae* would provide important insight into its ecological role and economic impact. The first step in that understanding is to characterize the morphological changes occurring during gall formation.

To gain insight into potential mechanisms of gall formation, we characterized how the galling insect *P. betae* reshapes the leaf morphology of the narrowleaf cottonwood, *P. angustifolia*, during the first stages of gall development. Using morphological and morphometric analyses, we characterized the alterations induced by the insect in the host plant during the first stages of gall development. We expected *P. betae* galls to (i) grow via cell hypertrophy and/or tissue hyperplasia, (ii) present an accumulation of nutrients in galled tissues as Larson and Whitham (1991) suggested as necessary to support the increasing demand of the growing colony of *P. betae*, and (iii) not display a true nutritive tissue as the aphid feeds on phloem contents.

## Material and methods

### Study system

**Fig. 1:**
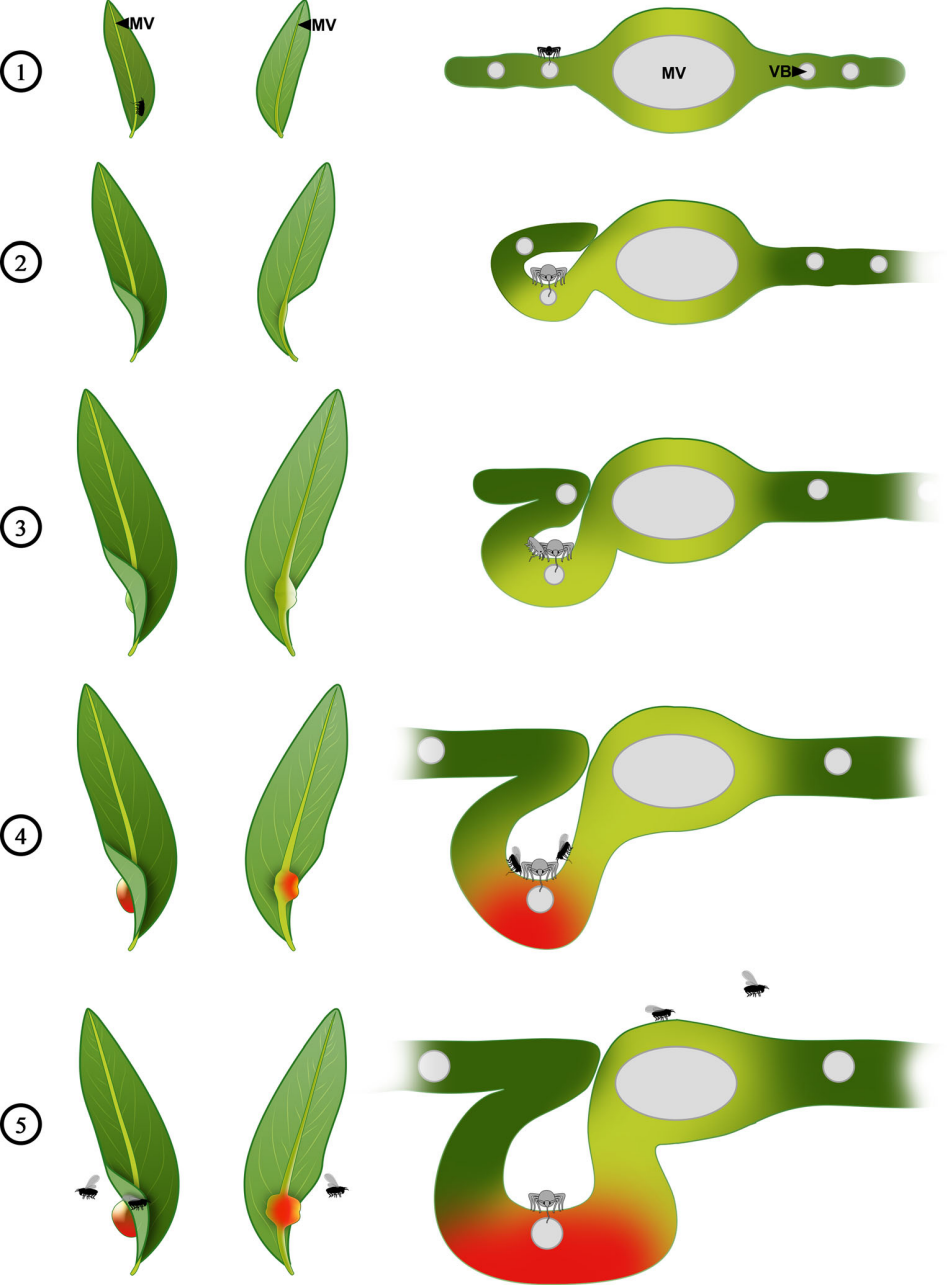
*Pemphigus betae* life cycle and gall ontogeny on its primary host plant, *Populus angustifolia*. Representations of galled leaves viewed from the upper/adaxial surface (left) and the lower/abaxial surface (center) of the leaf, as well as a cross-section (right), were compiled from the literature and results of this study (Harper 1959; Dunn 1960; Whitham 1979, 1980; Moran and Whitham 1988; Larson and Whitham 1991; Moran 1991; Whitham 1992; Wool 2004). *1*, In the spring, fundatrix nymphs emerge from overwintered eggs laid beneath the bark of poplar trees and colonize leaves just as the buds begin to open. Galls are initiated by a single stem mother along the midvein of an expanding leaf by probing the leaf tissue with her stylet moving between parenchyma cells to reach the phloem cells. *2*, Growth is arrested at the stylet insertion site, leading to the formation of a small depression on the leaf and causing the petiole to bend toward this area. *3*, Leaf tissue on one side of the midvein folds toward the center of the leaf and then back to create a fold on the abaxial side of the leaf. The fundatrix begins to parthenogenetically and viviparously produce a generation of several hundred (up to 300) apterate offspring. *4*, The wingless gall occupants become alate (winged). *5*, Alates disperse from the gall to deposit their larvae in the ground. These larvae colonize the roots of herbaceous secondary hosts where they feed during the summer without inducing galls. In the fall, alate sexuparae emerge from the roots, fly back to the poplar, and asexually produce sexual males and females. Their sexual reproduction produces a single egg (future fundatrix) in each female that is deposited in a tree bark crevice to overwinter. Structures not to scale for practical reasons. *MV* midvein with vascular bundles, *VB* vascular bundle

The sugarbeet root aphid *P. betae* (Hemiptera: Aphididae) induces galls on leaves of the narrowleaf cottonwood *P. angustifolia* (Salicaceae) (Harper 1959). The life cycle of *P. betae* in North America is an example of the holocycle (Fig. 1) (Moran and Whitham 1988). Each gall (size of mature galls: ∼25 mm long, ∼6 mm deep; Harper 1959) is induced by a single aphid (a fundatrix or stem mother) in the spring on their primary host poplar leaves (preferentially at their base) that are only a tenth to a quarter of their mature size (size of mature leaf: 50–90 mm long, 10–25 mm wide; USDA-NRCS 2008). Secondary hosts of *P. betae* include sugarbeets (from which the common name “sugarbeet root aphid” originates), carrots, turnips, Swiss chard, spinach, and lettuce, where they feed during the summer without inducing galls (Harper 1959; Moran and Whitham 1988; Larson and Whitham 1991; Moran 1991).

### Sample collection

Ungalled (control) and galled narrowleaf cottonwood leaves were collected in the field (Ogden Nature Center, Ogden UT, USA) in May 2008. We focused on developing leaves with young galls to study the first stages of gall development (Fig. 1, steps 3 and 4 before fundatrices start to reproduce). Leaves were 36.58 ± 0.78 mm long and 10.85 ± 0.26 mm wide with galls that were 5.96 ± 0.65 mm long, 1.73 ± 0.27 mm wide, and 1.41 ± 0.12 mm deep. Leaves and associated galling aphids were immediately fixed in 5 % glutaraldehyde in 50 mM sodium phosphate solution (pH 7). Excess leaf tissues were removed in the lab, and samples were cut to the appropriate size for further sectioning.

### Light microscopy

Leaf samples (*N* = 4 for controls and *N* = 5 for galls) were processed for light microscopy at IDEXX RADIL (BioResearch Laboratory, Columbia MO, USA). Samples were dehydrated in ethanol series, embedded in paraffin wax, and sectioned at 4–6 μm thick on a sliding microtome. Samples were stained with 0.5 % toluidine blue for 5 min, briefly rinsed in tap water, rinsed twice in 95 % alcohol for 1 min each, and rinsed twice in 100 % alcohol for 1 min each. Samples were then cleared in two changes of xylene and mounted on slides with mounting media and a coverslip (McManus and Mowry 1960). Toluidine blue was used to highlight general histological features. Images of ungalled control and galled tissues were acquired with a Leica 5500B light microscope (Leica Microsystems, Germany) equipped with a Leica DFC290 camera and the Leica Application Suite v.4.6.0 software at the Molecular Cytology Core, University of Missouri (Columbia MO, USA).

### Transmission electron microscopy

Leaf samples (*N* = 5 for controls and *N* = 8 for galls) were processed for transmission electron microscopy (TEM) at the Electron Microscopy Core Facility (University of Missouri, Columbia MO, USA). Unless otherwise stated, all reagents were purchased from Electron Microscopy Sciences (Hatfield PA, USA). Tissues were fixed in 5 % glutaraldehyde in 50 mM sodium phosphate solution (pH 7), then rinsed with 100 mM sodium cacodylate buffer (pH 7.35) containing 10 mM 2-mercaptoethanol (Sigma Aldrich, St. Louis MO, USA) and 130 mM sucrose (further referred to as 2-ME buffer). Secondary fixation was performed with 1 % osmium tetroxide in 2-ME buffer using a Pelco Biowave (Ted Pella Inc., Redding CA, USA) operated at 100 W for 1 min. Specimens were then incubated at 4 °C for 1 h, rinsed with 2-ME buffer followed by distilled water. Samples were then dehydrated using the Pelco Biowave, a graded ethanol dehydration series (per exchange, 100 W for 40 s), transitioned into acetone, and then infiltrated with Epon/Spurr’s resin (250 W for 3 min) and polymerized at 60 °C overnight. Next, embedded tissue was cut to a thickness of 85 nm using a microtome (Ultracut UCT, Leica Microsystems, Germany) and a diamond knife (Diatome, Hatfield PA, USA). These sections were post-stained with Reynolds lead citrate stain (Reynolds 1963) and 5 % aqueous uranyl acetate. Images of cells from upper epidermis, palisade parenchyma, and spongy parenchyma (first few layers) of ungalled control and galled tissues were acquired with a JEOL JEM 1400 transmission electron microscope (JEOL, Peabody MA, USA) at 80 kV on a Gatan Ultrascan 1000 CCD (Gatan Inc., Pleasanton CA, USA).

### Image analysis

**Fig. 2:**
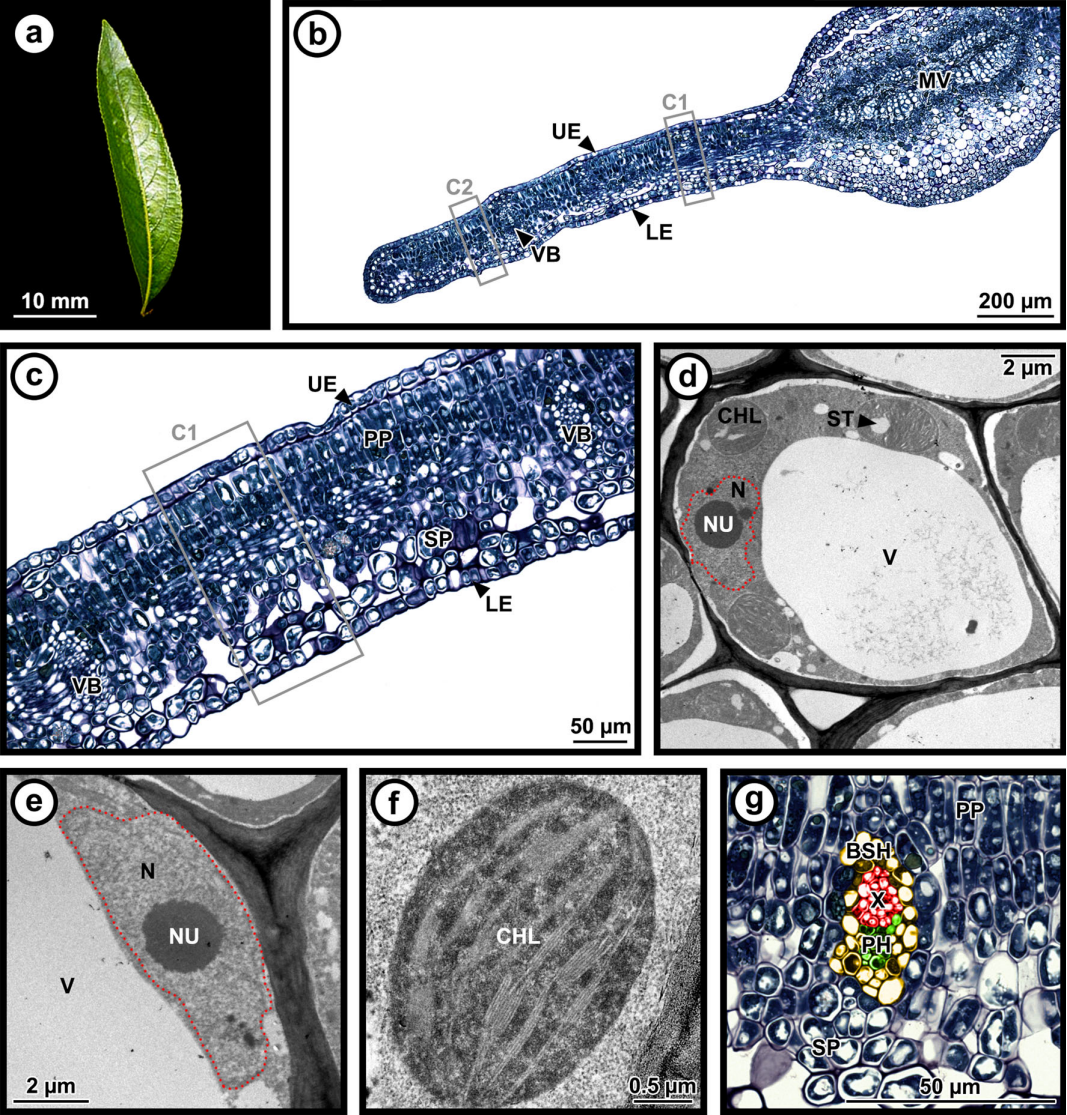
Morphology of ungalled control poplar *Populus angustifolia* leaves. Ungalled leaf (**a**). The unaltered leaf (**b**, **c**) has one layer of upper epidermis, two layers of palisade parenchyma, spongy parenchyma with large intercellular spaces, and one layer of lower epidermis. Cells generally contain only one large vacuole (**d**), a nucleus, and a visible nucleolus (**e**), intact chloroplasts (**f**), and functional vascular bundles (**g**). C1 = region of interest for cell counts on ungalled control tissues, C2 = internal control for cell counts on ungalled control tissues. The *red dotted line* outlines the nucleus. False colors: red = xylem, green = phloem, yellow = bundle sheath cells. T.E.M. images (**d**, **e**, **f**) from the spongy parenchyma of the C1 region, with the exception of vascular bundles (**g**) that are from a secondary vein in the palisade parenchyma of the C1 region. *BSH* bundle sheath cells, *CHL* chloroplast, *LE* lower epidermis, *MV* midvein with vascular bundles, *N* nucleus, *NU* nucleolus, *PH* phloem, *PP* palisade parenchyma, *SP* spongy parenchyma, *ST* starch, *UE* upper epidermis, *V* vacuole, *VB* vascular bundle, *X* xylem

**Fig. 3:**
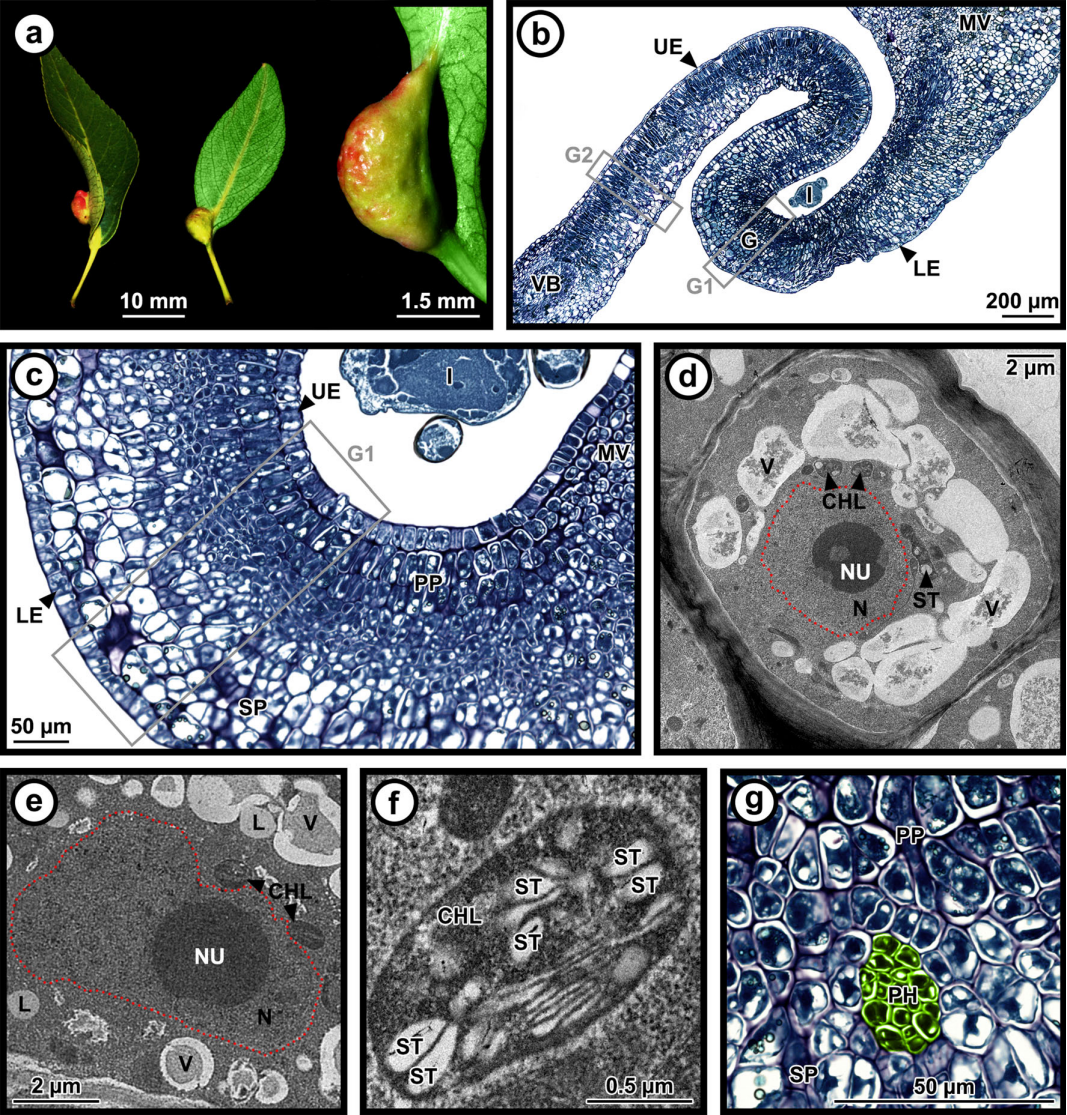
Morphology of young galls induced by *Pemphigus betae* on poplar *Populus angustifolia* leaves. Galled leaves (**a**) viewed from the upper/adaxial surface (left) and the lower/abaxial surface (center) of the leaf, as well as a gall viewed from the side (right). Leaf tissue on one side of the midvein bends towards the center of the leaf and then back to create a fold that surrounds the aphid on the abaxial side of the leaf (**b**). The fold then grows abaxially through periclinal and anticlinal divisions, effectively eliminating intercellular spaces from the spongy parenchyma (**c**). Cells on the adaxial surface are more numerous and smaller than cells near the abaxial surface are, creating the large fold that surrounds the insect. The insect induces altered cells within the gall. In galled tissues, cells (**d**) are generally smaller, with reduced and fragmented vacuoles, larger nucleoli and nuclei (**e**), and smaller and more numerous chloroplasts (**f**) than their ungalled tissue counterparts (see Fig. 2). Vascular bundles (**g**) are comprised solely of phloem. G1 = region of interest for cell counts on galled tissues, G2 = internal control for cell counts on galled tissues. The *red dotted line* outlines the nucleus. False colors: green = phloem. T.E.M. images (**d**, **e**, **f**) are all taken in the spongy parenchyma of the G1 region, except images of vascular bundles (**g**) that have been taken on a secondary vein in the palisade parenchyma of the G1 region. *CHL* chloroplast, *G* gall, *I* insect, *L* lipid, *LE* lower epidermis, *MV* midvein, *N* nucleus, *NU* nucleolus, *PH* phloem, *PP* palisade parenchyma, *SP* spongy parenchyma, *ST* starch, *UE* upper epidermis, *V* vacuole, *VB* vascular bundle

Light and TEM images were analyzed with the ImageJ version 1.49 m software (National Institutes of Health, USA) and the Fiji plugin. Thirty-six light microscopy images from nine samples (*N* = 4 for controls and *N* = 5 for galls) were used for counting cells in a 100-μm wide transect from adaxial to abaxial surfaces and for measuring gall/leaf thickness. Cell number was determined for each tissue type (upper epidermis, palisade parenchyma, spongy parenchyma, lower epidermis, and vascular bundle) and expressed as cell number per tissue type in the 100-μm transect. Cell density was determined by dividing the number of cells in each tissue type by the area of this specific tissue type within the 100-μm wide transect, and expressed as cell number per micrometers squared. On each image, we analyzed the region of interest near the midvein (C1 for ungalled control tissues or G1—the gall—for galled tissues) and another zone between the midvein and the leaf margin as an internal control (C2 for ungalled control tissues or G2 for galled tissues) (see Figs. 2b and 3b).

For morphometric analyses in C1 (control, see Fig. 2b, c) and G1 (gall, see Fig. 3b, c) zones, we focused on the upper epidermis, palisade parenchyma, and first half of the spongy parenchyma (upper/adaxial portion) as it is closest to the gall interior in which the fundatrix and nymphs feed. Morphometric data is therefore unavailable for the second half of the spongy parenchyma (lower/abaxial portion) and the lower epidermis.

Ninety-eight cells from electron micrographs of 13 samples (*N* = 5 for controls and *N* = 8 for galls) were acquired from C1 (control, see Fig. 2b, c) and G1 (gall, see Fig. 3b, c) zones only and used for cell morphometry (length, width, area, and number of cells and organelles: nucleus, nucleolus, vacuoles, chloroplasts, mitochondria, and starch granules and lipid droplets). Only cell and organelle numbers and average sizes (area expressed as μm^2^) are presented here. Ratios between nucleus and cell size, as well as between nucleolus and nucleus were calculated from cell and organelle areas. TEM images taken included upper epidermis, palisade parenchyma, and the first few layers of the spongy parenchyma (upper/adaxial portion) of C1 and G1 zones. Thin sections were not taken of the second half of the spongy parenchyma (lower/abaxial portion) or the lower epidermis.

Cell organelles, starch granules, and lipid droplets were identified according to the literature (Bronner 1992; Oliveira et al. 2010, 2011; Vecchi et al. 2013; Carneiro and Isaias 2015a). Nutrient contents (starch granules and lipid droplets) of each analyzed cell were estimated by summing the area of each nutrient allowing comparison between ungalled control and galled tissues for each tissue type.

### Statistical analyses

Statistical analyses were performed using R version 2.13.1 and RStudio version 0.98.1103 (The R Foundation for Statistical Computing, Vienna, Austria). Cell number and density in C1, C2, G1, and G2 zones (see Figs. 2b and 3b) were compared using either one-way ANOVA or the Kruskal–Wallis tests, depending on whether the data for the specific comparison had a normal or non-normal distribution). Where significant effects were observed, post hoc comparisons were performed using Tukey HSD test and Mann–Whitney test with Bonferroni correction, depending on the normality of the data. Cell number is presented as cell number per tissue type in a 100-μm wide transect and cell density as cell number per micrometers squared for each tissue type in the 100-μm transect (average ± S.E.M.). Lengths, widths, and areas of cells and organelles and total areas of starch granules and lipid droplets in ungalled control (zone C1, see Fig. 2b, c) and galled (zone G1, see Fig. 3b, c) tissues (Table 1) were compared using Student and Welch *t* test (normal distribution) and Mann–Whitney test (non-normal distribution). The level of significance used in all tests was *p* value ≤0.05. All measurements are presented as micrometers and areas as micrometers squared (average ± S.E.M.).

**Table 1:** Morphometric measurements and counts of cells and organelles

	Control	Gall	Control	Gall
Cell	Average size (μm^2^)			
UE	163.69 ± 17.92a	94.61 ± 9.52b		
PP	154.16 ± 13.68a	130.04 ± 15.29b		
SP	219.16 ± 14.97a	183.56 ± 11.39b		
Nucleus	Average size (μm^2^)		Ratio nucleus/cell	
UE	10.92 ± 1.99a	15.82 ± 1.74a	0.07 ± 0.01a	0.17 ± 0.02b
PP	15.95 ± 2.83a	19.79 ± 2.55a	0.09 ± 0.01a	0.15 ± 0.02b
SP	11.99 ± 3.63a	16.38 ± 1.91a	0.06 ± 0.02a	0.10 ± 0.01a
Nucleolus	Average size (μm^2^)		Ratio nucleolus/nucleus	
UE	2.75 ± 0.71a	4.04 ± 0.46a	0.20 ± 0.03a	0.23 ± 0.02a
PP	5.57 ± 1.00a	3.06 ± 0.62a	0.30 ± 0.05a	0.19 ± 0.02b
SP	1.70 ± 0.59a	4.80 ± 0.75a	0.27 ± 0.12a	0.33 ± 0.04a
Vacuole	Average size (μm^2^)		Number/cell	
UE	19.91 ± 5.81a	2.78 ± 0.43a	6.00 ± 1.15a	13.42 ± 1.06b
PP	22.96 ± 5.81a	4.20 ± 0.76b	5.29 ± 0.97a	11.41 ± 0.97b
SP	75.69 ± 20.84a	20.07 ± 4.31b	2.82 ± 0.80a	6.24 ± 1.59a
Mitochondrion	Average size (μm^2^)		Number/cell	
UE	0.15 ± 0.02a	0.13 ± 0.02a	2.75 ± 0.85a	3.00 ± 1.13a
PP	0.18 ± 0.04a	0.33 ± 0.10a	2.00 ± 0.58a	1.00 ± 0.00a
SP	0.31 ± 0.03a	0.30 ± 0.07a	6.60 ± 2.06a	2.00 ± 0.00a
Chloroplast	Average size (μm^2^)		Number/cell	
UE	0.81 ± 0.12a	0.58 ± 0.04a	3.91 ± 0.92a	8.22 ± 1.39b
PP	2.33 ± 0.19a	1.18 ± 0.06b	6.23 ± 0.87a	11.85 ± 1.61b
SP	2.55 ± 0.19a	1.32 ± 0.09b	10.40 ± 2.69a	6.17 ± 0.97a
Starch	Total area (μm^2^)			
UE	0.17 ± 0.00a	0.56 ± 0.14a		
PP	0.24 ± 0.14a	2.13 ± 0.89a		
SP	0.73 ± 0.34a	2.33 ± 0.56b		
Lipid	Total area (μm^2^)			
UE	0.56 ± 0.19a	2.54 ± 1.08a		
PP	0.58 ± 0.10a	0.94 ± 0.23a		
SP	0.93 ± 0.32a	1.43 ± 0.83b		

Measurements and numbers of cells and organelles for each tissue type (upper epidermis, palisade parenchyma, and spongy parenchyma) in ungalled control (zone C1, see Fig. 2b, c) and galled tissues (zone G1, see Fig. 3b, c) from electron micrographs. Statistical differences (*p* value ≤ 0.05) between means for ungalled controls and galls in each tissue type (comparisons between columns: control vs. gall for a same line) are shown by different letters (a, b). Data shown as average ± S.E.M

*PP* palisade parenchyma, *SP* spongy parenchyma, *UE* upper epidermis

## Results

### Morphology of ungalled and galled tissues

From the adaxial surface to the abaxial surface, ungalled leaves (36.58 ± 0.78 mm long and 10.85 ± 0.26 mm wide; Fig. 2a) typically had a single cell layer of upper epidermis, two cell layers of palisade parenchyma with small intercellular spaces, three “layers” of spongy parenchyma (about twice as thick as the palisade parenchyma) with large intercellular spaces, and a single layer of lower epidermis in C1 and C2 zones (Fig. 2b, c).

**Fig. 4:**
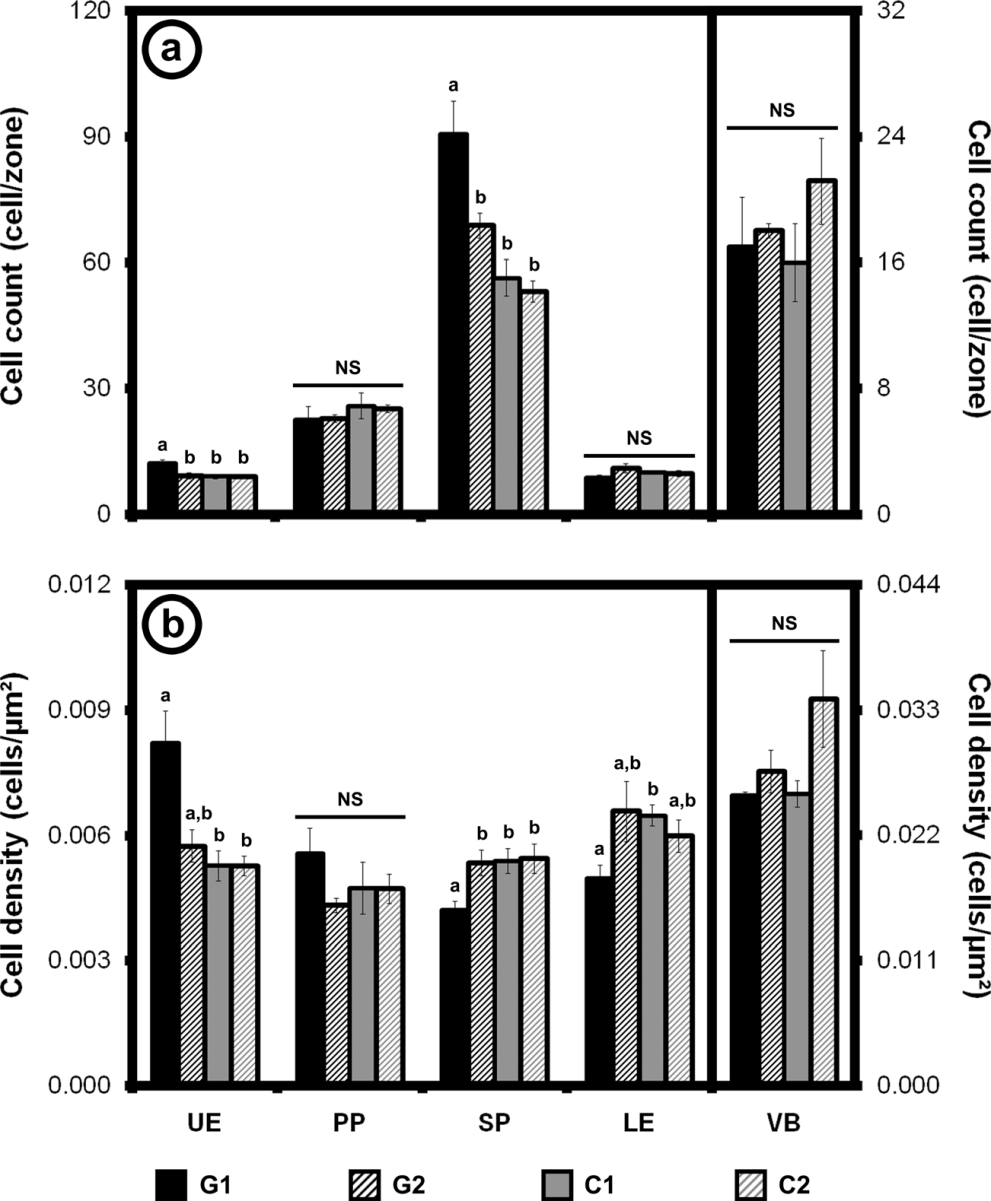
Cell numbers and densities. Cell numbers (**a**) and densities (**b**) for each tissue type in ungalled controls and galls from light micrographs. Statistical differences (*p* value ≤ 0.05) between means for ungalled controls and galls in each tissue type are shown by different letters (**a**, **b**). Data shown as average ± S.E.M. C1 = region of interest for cell counts on ungalled control tissues, C2 = internal control for cell counts on ungalled control tissues, G1 = region of interest for cell counts on galled tissues, G2 = internal control for cell counts on galled tissues (see Figs. 2b and 3b). *LE* lower epidermis, *PP* palisade parenchyma, *SP* spongy parenchyma, *UE* upper epidermis, *VB* vascular bundle

On galled leaves (galls 5.96 ± 0.65 mm long, 1.73 ± 0.27 mm wide, and 1.41 ± 0.12 mm deep; Fig. 3a), leaf tissue on one side of the midvein was folded toward the center of the leaf and then back to create a fold on the abaxial side of the leaf (Figs. 1 and 3b). In the G1 zone, periclinal and anticlinal cell division was evident between the palisade layer and spongy parenchyma, effectively eliminating intercellular spaces from the spongy parenchyma (Fig. 3c). Cells on the adaxial surface of the G1 zone were more numerous and smaller than cells near the abaxial surface were (density 0.00820 cell/μm^2^ in the upper epidermis vs. 0.00496 cell/μm^2^ in the lower epidermis; Welch *t* test, *p* value = 0.002) (Figs. 2c, 3c, and 4ab, Table 1), creating the large fold that surrounded the insect.

The leaf was thicker in the gall (G1 = 312.34 ± 9.05 μm) than in the control zone on the same leaf (G2 = 239.97 ± 10.40 μm; Mann–Whitney post hoc test with Bonferroni correction, *p* value < 0.001; Fig. 3b), whereas the control regions on ungalled leaves, C1 and C2, did not differ (C1 = 207.50 ± 5.25 μm, C2 = 207.37 ± 6.67 μm; Mann–Whitney post hoc test with Bonferroni correction, *p* value = 0.442; Fig. 2b). The largest difference in thickness between ungalled control and galled tissues came from the spongy parenchyma which exhibited a +104.39 % increase in the gall. In galled tissues, cell size and density varied with cell type (Figs. 2b, c, 3b, c, and 4a; Table 1). The cell density was higher for the upper epidermis and the palisade parenchyma and lower for the spongy parenchyma and the lower epidermis compared to the control (Tukey HSD post hoc test and Mann–Whitney post hoc test with Bonferroni correction, *p* value < 0.05) (Fig. 4b). Two different regions can be distinguished in the gall: the upper/adaxial portion with cells that are smaller and more numerous, and the lower/abaxial portion with cells that are bigger and less numerous (Fig. 3c).

In ungalled tissues, vascular bundles were comprised of xylem located toward the adaxial side of the leaf with phloem located toward the abaxial side, and the entire bundle was surrounded by bundle sheath cells (Fig. 2g). In galled tissues, the vascular bundles were disorganized; xylem and bundle sheath cells were absent. The stylet pathway between parenchyma cells to reach the phloem was not observed on the sections of the galls that were analyzed for this study. Phloem sieve tube elements and companion cells were more numerous and larger in galled tissues than in ungalled tissues (Figs. 2g and 3g).

### Morphometry of cells and organelles in ungalled and galled tissues

Nuclei, as well as nucleoli, in the galls and controls were of the same size (Figs. 2d, e and 3d, e, Table 1); however, cells were smaller in the gall (upper epidermis, palisade parenchyma, upper/adaxial portion of the spongy parenchyma), leading to a higher ratio between the nuclei and their respective cells in the upper epidermis and palisade parenchyma (Figs. 2e and 3e, Table 1). Mitochondria size was also similar in galls and controls. Vacuoles were reduced and fragmented in cells from each tissue type of galled tissues but were intact and of normal size in ungalled tissues (Figs. 2d and 3d, Table 1). Chloroplasts were smaller in the gall than in the control tissues, especially in the palisade and spongy parenchyma. Chloroplasts were also more numerous in the upper epidermis and palisade parenchyma but tended to be less numerous in the spongy parenchyma (Table 1). Electron microscopy revealed that chloroplasts in the control tissues had normal grana with flattened thylakoid membranes and a more rounded shape, whereas chloroplasts in galled tissues are smaller, had degraded, having lost their round shape and organization, and exhibited underdeveloped lamellation, disorganized grana stacks, and swollen thylakoids (Figs. 2f and 3f).

Starch content, as estimated by structural features, was 3–10-fold higher in all of the galled tissues compared to all of their respective control tissues, although the difference was only statistically significant in the spongy parenchyma (Table 1). Cells in the palisade and spongy parenchyma contained more starch than those in the upper epidermis did (Figs. 2f and 3f, Table 1). Lipid content, as estimated by structural features, also tended to be greater in all of the galled tissues (Table 1).

## Discussion

**Fig. 5:**
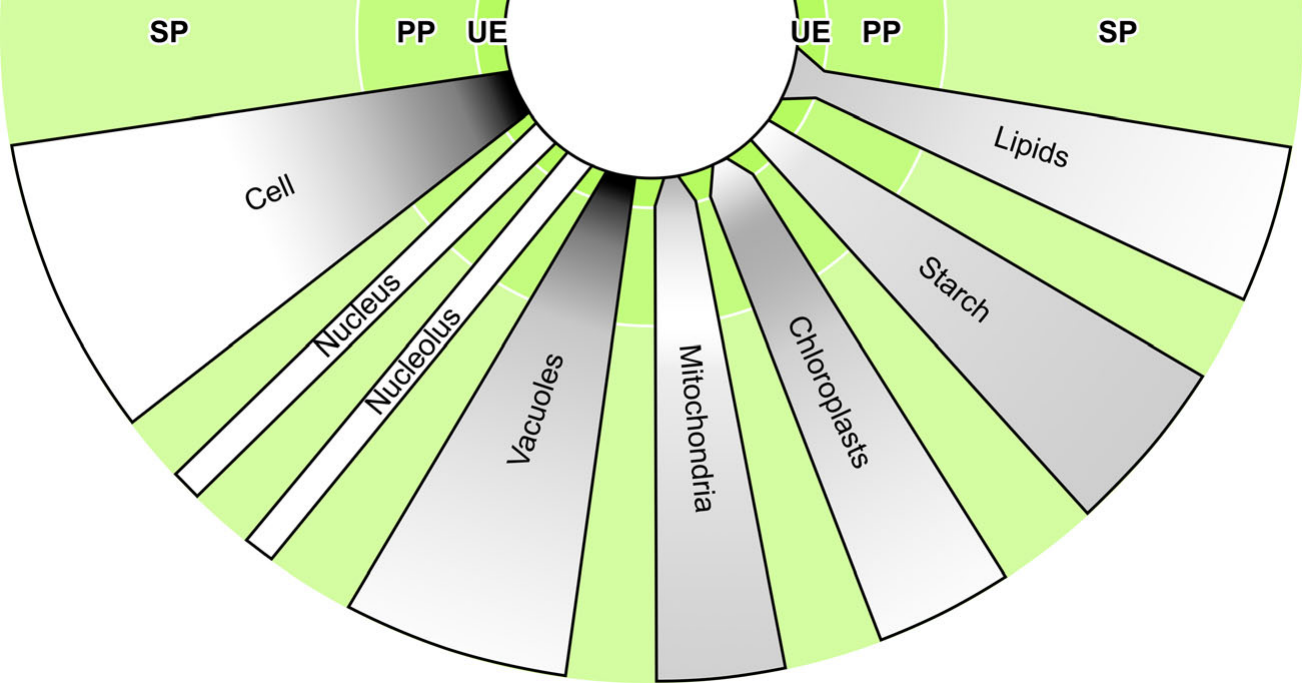
Representation of metabolic activities and cell/organelle size and shape within the nutritive tissues of *Pemphigus* galls. Average size of the cells/organelles in each tissue is represented by the width of the ray, and the gradient of the number/density is represented by *shading*. Wider areas are indicative of larger cells, organelles, and nutrients while thinner areas are indicative of smaller ones. *Darker shades of gray* are equivalent to higher numbers/densities, while *lighter shades of gray* are equivalent to lower numbers/densities of cells, organelles, and nutrients. Different averages do not necessarily reflect statistically significant differences (see Fig. 4 and Table 1 for statistical analysis). Shape and color are relative to the item they represent and not to be compared with others. The *green background* represents the gall. *PP* palisade parenchyma, *SP* spongy parenchyma, *UE* upper epidermis. Adapted from Bronner (1992)

The morphological analysis of *P. betae* galls performed in this study describes for the first time how this insect alters leaf morphology and structure (Fig. 1) to create a leaf fold gall (Rohfritsch 1992) and quantifies of the extent of the modifications. The fundatrices insert their stylet between cells into the leaf tissue on one side of the midvein to reach the phloem sap from vascular bundles in the first few layers of spongy parenchyma of the gall (Fig. 1, step 1). As a result, growth is arrested at the stylet insertion site, a small depression forms, and the petiole bends toward the center and then back again to create the gall (Fig. 1, steps 2 and 3). Growth continues as normal on the opposite side of the petiole. The tissue changes in the galled leaf were caused by tissue hyperplasia (more numerous cells) in the upper/adaxial portion of the mesophyll and cell hypertrophy (bigger cells) in the lower/abaxial portion of the gall (Figs. 3c and 5). Growth via tissue hyperplasia and cell hypertrophy is a common feature in galls and pseudogalls (when the insect is not enclosed within plant tissue, i.e., open galls; Zhang and Chen 1999) induced by different aphids and relatives such as *Phloeomyzus passerinii*, *Eriosoma lanigerum*, *Adelges laricis*, *Adelges abietis*, and *Daktulosphaira vitifoliae*, but also in other insect-induced galls in general (Elzen 1983; Brown et al. 1991; Rohfritsch and Anthony 1992; Wool and Bar-El 1995; Forneck et al. 2002; Kraus et al. 2002; Arduin et al. 2005; Álvarez et al. 2009; Oliveira and Isaias 2010a; Carneiro et al. 2014; Dardeau et al. 2014a, b; Tooker and Helms 2014; Carneiro et al. 2015; Kurzfeld-Zexer et al. 2015; Suzuki et al. 2015). Cell divisions occur in several planes, increasing the number of cell layers and the thickness of the parenchyma, and are related to the new functions of the mesophyll as a feeding site and a protective barrier for the insect (Mani 1964; Rohfritsch 1992; Moura et al. 2008; Dias et al. 2013; Carneiro et al. 2015).

As phloem-feeders, aphids must have access to the vascular system of the host plant or induce the formation of new vascular elements at the galling sites as in galls induced by *Geoica wertheimae* aphids (Wool et al. 1999; Wool 2005). The modified structure of vascular bundles observed in this study is a key feature of gall development. The absence of xylem and bundle sheath cells may facilitate the aphid’s access to its nutrient source in the phloem, thus eliminating the need for stylet penetration into or around these cells (Figs. 3g and 4b). Many galling insects induce and feed on a nutritive layer within the gall that accumulates nutrients. As with other aphids and psyllids, *P. betae* feed directly from the phloem and therefore no true nutritive tissue was expected to develop (Bronner 1992; Álvarez et al. 2009; Álvarez 2011; Kurzfeld-Zexer et al. 2015). However, *P. betae* does affect the size and shape of cells and organelles, some of which exhibit features typical of nutritive cells induced by other galling insects and root-knot nematodes, including a conspicuous nucleolus, a fragmented vacuole, smaller and degraded chloroplasts, and a dense cytoplasm (Jones and Payne 1978; Bronner 1992; Isaias and Oliveira 2012; Rodiuc et al. 2014). We refer here to those morphological changes as nutritive-like because they do not include the larger nucleus and more numerous mitochondria present in true nutritive layers (Fig. 5).

Cytological and histochemical gradients have also been reported in galls induced by other sucking insects, such as in galls induced by two psyllid species, *Euphalerus ostreoides* and *Nothotrioza cattleiani*, and three aphid species, *Paracletus cimiciformis*, *Forda marginata*, and *Forda formicaria*, (Álvarez et al. 2009; Oliveira and Isaias 2010b; Isaias and Oliveira 2012; Carneiro and Isaias 2015a, b). In the first stages of gall development we examined, cells exhibited large nuclei resembling those reported for metabolically active cells in the nutritive tissues of galls induced by Thysanoptera (Raman and Ananthakrishnan 1983; Carneiro and Isaias 2015a). Another feature of nutritive tissue is starch accumulation in the vicinity of hypertrophied cells, which provides soluble sugars to the insect (Bronner 1992; Rohfritsch and Anthony 1992; Forneck et al. 2002). An accumulation of nutrients (starches and to a lesser extent lipids) also occurs in the palisade and first few layers of spongy parenchyma of the *P. betae* gall but not to the extent as that observed in nutritive cells of galls induced by Cecidomyiidae, Cynipidae, and a microlepidopteran species (Bronner 1992; Rohfritsch 1992; Oliveira et al. 2010; Vecchi et al. 2013). Larson and Whitham (1991) used ^14^C-labeling experiments to characterize the alteration of the source-sink translocation patterns in poplar infected by *P. betae* aphids. This study showed that *P. betae* galls function as physiological sinks, drawing in resources from the surrounding plant tissue (galled and surrounding leaves) leading to an accumulation of nutrients at the feeding site. The imported nutrients improve the nutritional quality of phloem sap and accumulate in the most internal cell layers (Larson and Whitham 1991; Inbar et al. 1995; Fay et al. 1996; Koyama et al. 2004; Suzuki et al. 2009; Dias et al. 2013). Thus, even though the *Pemphigus* aphids do not consume the gall tissue, they induce changes in the plant vascular system that lead to nutrient accumulation in surrounding galled tissues. The capacity of aphids to alter the host plant to create food sources of a higher quality than in ungalled plants likely enables the hundreds of offspring to feed for several weeks in a small and confined space before dispersion (Larson and Whitham 1991; Wool et al. 1998; Isaias and Oliveira 2012). Indeed, Larson and Whitham (1991) showed that aphids respond to the increasing food demands of a growing colony in mature galls by importing more resources from neighboring leaves. It appears that the galling aphid effectively manipulates plant morphogenesis to produce new cell fates that increase the adaptive value of the gall structure (Stone and Schönrogge 2003; Carneiro and Isaias 2015a).

## Conclusion

*P. betae* feeding on the narrowleaf cottonwood *P. angustifolia* induces of a leaf fold gall formation. In the first stages of gall development, we observed cell hyperplasia and hypertrophy, consistent with a model of gall formation by alteration of insect-induced or insect-supplied phytohormones. The long and intimate relationship of endophagous feeding insects with their host plants likely facilitates biochemical and hormonal crosstalk between insects and plants, resulting in host plant manipulation by insects (Schultz 2002; Schultz and Appel 2004; Body et al. 2013; Giron et al. 2013, 2015; Giron and Glevarec 2014; Robischon 2015). Whether homologous or convergent, shared signaling systems provide herbivores with the ability to generate optimal microenvironments for growth, survival, and reproduction.
